# Efficacy of oblique lumbar interbody fusion versus transforaminal lumbar interbody fusion in the treatment of lumbar degenerative diseases: a systematic review and meta-analysis

**DOI:** 10.1007/s00402-023-04880-4

**Published:** 2023-04-20

**Authors:** Xi-yong Li, Yun-lu Wang, Su Yang, Chang-sheng Liao, Song-feng Li, Peng-yong Han, Peng-fei Han

**Affiliations:** 1 Department of Orthopaedics, Heping Hospital Affiliated to Changzhi Medical College, Changzhi, People’s Republic of China; 2 Graduate School, Changzhi Medical College, 110 South Yan’an Road, Changzhi, 046000 People’s Republic of China

**Keywords:** Lumbar degenerative diseases, OLIF, TLIF, Meta

## Abstract

**Introduction:**

This meta-analysis aimed to compare the differences in postoperative efficacy between oblique lumbar interbody fusion (OLIF) and transforaminal lumbar interbody fusion (TLIF) in the treatment of lumbar degenerative diseases.

**Materials and methods:**

Strictly based on the search strategy, we searched the published papers on OLIF and TLIF for the treatment of lumbar degenerative diseases in PubMed, Embase, CINAHL, and Cochrane Library. A total of 607 related papers were retrieved, and 15 articles were finally included. The quality of the papers was evaluated according to the Cochrane systematic review methodology, and the data were extracted and meta-analyzed using Review manager 5.4 software.

**Results:**

Through comparison, it was found that in the treatment of lumbar degenerative diseases, the OLIF group had certain advantages over the TLIF group in terms of intraoperative blood loss, hospital stay, visual analog scale (VAS) for leg pain (VAS-LP), Oswestry disability index (ODI), disc height (DH), foraminal height (FH), fused segmental lordosis (FSL), and cage height, and the differences were statistically significant. The results were similar in terms of surgery time, complications, fusion rate, VAS for back pain (VAS-BP) and various sagittal imaging indicators, and there was no significant difference.

**Conclusions:**

OLIF and TLIF can relieve low back pain symptoms in the treatment of lumbar degenerative diseases, but OLIF has certain advantages in terms of ODI and VAS-LP. In addition, OLIF has the advantages of minor intraoperative trauma and quick postoperative recovery.

## Introduction

Low back pain is one of the most common health diseases. According to incomplete statistics, approximately one-third of the population suffers from low back pain yearly. Research shows that the incidence of low back pain increases with age up to 60 years and decreases after that [1]. Lumbar disc herniation, lumbar spinal stenosis, spondylolisthesis, and other lumbar degenerative diseases are the main causes of low back pain [2]. Chronic low back pain caused by lumbar degenerative diseases severely affects the daily life of patients and even causes a huge economic burden on society and individuals. In some countries, lower back pain treatment costs three times that of cancer [3]. Currently, the treatment options for low back pain caused by lumbar degenerative diseases mainly include conservative and surgical treatments. Conservative treatment is primarily based on physical therapy combined with multimodal analgesia. However, some studies have shown that only approximately half of the patients undergoing conservative treatment have significant improvement in the symptoms of low back pain in the short term, while surgical treatment can not only relieve pain in a short time but also control pain symptoms well within 4 years after surgery [4]. Chen et al. found that surgical treatment has advantages in terms of short-term pain and quality of life compared with conservative treatment [5], and other scholars believe that early surgical treatment of symptomatic lumbar degenerative diseases is conducive to faster recovery of neurological function [2]. Traditional surgical approaches include posterior lumbar interbody fusion (PLIF) and transforaminal lumbar interbody fusion (TLIF). Some studies have shown that TLIF has lower postoperative complications and intraoperative trauma when its clinical efficacy is roughly equivalent [6]. Although TLIF has a good intervertebral fusion rate, its posterior approach is prone to damage the spinal canal, nerve roots, and posterior tension bands [7]. In 2012, Silvestre reported an oblique lumbar interbody fusion (OLIF) approach from the space between the psoas muscle and the abdominal aorta. This operation avoids the destruction of the posterior structure and postoperative pain and fatigue of the lumbar muscles because it does not damage the proximal nerve trunk of the lumbar muscles [8]. However, some studies have also shown that OLIF can damage the sympathetic nerve and venous structure of the anterior psoas muscle [9]. Therefore, this study aimed to compare the therapeutic effects of OLIF and TLIF in the treatment of lumbar degenerative diseases in terms of intraoperative trauma, postoperative pain relief, postoperative functional recovery, and complications to provide a theoretical basis and help in clinical work.

## Materials and methods

### Search strategy

Search databases, such as PubMed, Embase, CINAHL, and Cochrane Library, were searched according to the search strategy. The language of the literature, sample size, and age of the participants in the literature were not within limits. Relevant literature in all databases was searched, and the searched keywords were OLIF, oblique lumber interbody fusion, TLIF, transforaminal lumbar interbody fusion, and lumbar disease. The search strategy was {[(oblique lumber interbody fusion) OR (OLIF)] OR [(TLIF) OR (transforaminal lumbar interbody fusion)]} AND (degenerative disease of the lumbar spine).

### Surgical techniques

#### OLIF

The patient is placed in the right-lying position, an inclined cutaneous incision is made in the left extra-abdominal area, the myofascia is opened, and the muscle is bluntly separated. Retroperitoneal dissection with the ventral margin of the psoas muscle and exposure of the intervertebral disc space by transverse pushing over the psoas muscle is performed. The disc space is prepared, and a fusion device is placed with sufficient height and angle. Finally, no additional internal fixations are performed.

#### TLIF

The patient is placed in the prone position, approached through the dorsal median incision, the paraspinal muscles are peeled off, exposing the lamina and facet joints of the surgical segment, inserting pedicle screws, the disc after unilateral facet joint resection is cleared, and a suitable cage is placed through the intervertebral foramen. A longitudinal rod is placed and fixed using pressurized screws.

### Inclusion and exclusion criteria

The inclusion criteria were as follows: (1) research participants: patients diagnosed with lumbar degenerative diseases, such as lumbar disc herniation, lumbar spinal stenosis, and lumbar spondylolisthesis; if there was no significant relief of symptoms, such as low back pain after 6 months of regular conservative treatment, surgery was considered, and all surgeries were single-segment surgery; (2) interventions: experimental (OLIF) and control groups (TLIF); (3) outcome indicators: surgery time, blood loss, hospital stay, VAS, ODI, DH, FH, FSL, cage height, fusion rate, complications, and imaging indicators, including 12 items; (4) type of included study design: a clinical controlled trial.

The exclusion criteria were as follows: (1) conservative treatment of lumbar degenerative diseases; (2) reviews, systematic reviews, case reports, letters, and repeated publications; (3) noncase-control studies; and (4) literature with incomplete or irrelevant outcome indicators.

### Data extraction and literature quality assessment

Two independent researchers extracted data separately using a strict standard protocol, and disagreements were resolved by discussion or joint evaluation with more senior researchers until a consensus was reached. Participant selection, comparability between the groups, and outcome measures were scored by two independent persons according to the Newcastle–Ottawa scale (NOS) for all final inclusion studies. The higher the total score, the higher the quality of the study. In the outcome measure items, a follow-up time > 1 year and a loss-to-follow-up rate < 5% were scored separately. The NOS score was divided into three grades: low, medium, and high quality, namely, < 5, 5–7, and 8–9 points.

### Statistical methods

Meta-analysis was performed on the data extracted from the included literature using the Review manager 5.4 software. According to the odds ratio (OR) and 95% confidence interval (CI) of the results, dichotomous variables were expressed, and continuous variables were represented by mean difference or standard mean difference and 95% (CI). Differences were considered statistically significant at *P* ≤ 0.05. If *I*^2^ ≤ 50% among the study groups, it indicated no significant statistical heterogeneity, and a fixed-effects model was used for pooled analysis. If *I*^2^ > 50%, there was a large heterogeneity between the studies. Subgroup analysis and sensitivity analysis were performed to find the source of heterogeneity and try to eliminate the heterogeneity; however, when the heterogeneity could not be eliminated, a random-effects model was used. The report was performed according to the preferred reporting items for systematic reviews and meta-analyses guidelines.

## Result

### Essential features of the included literature

**Fig. 1:**
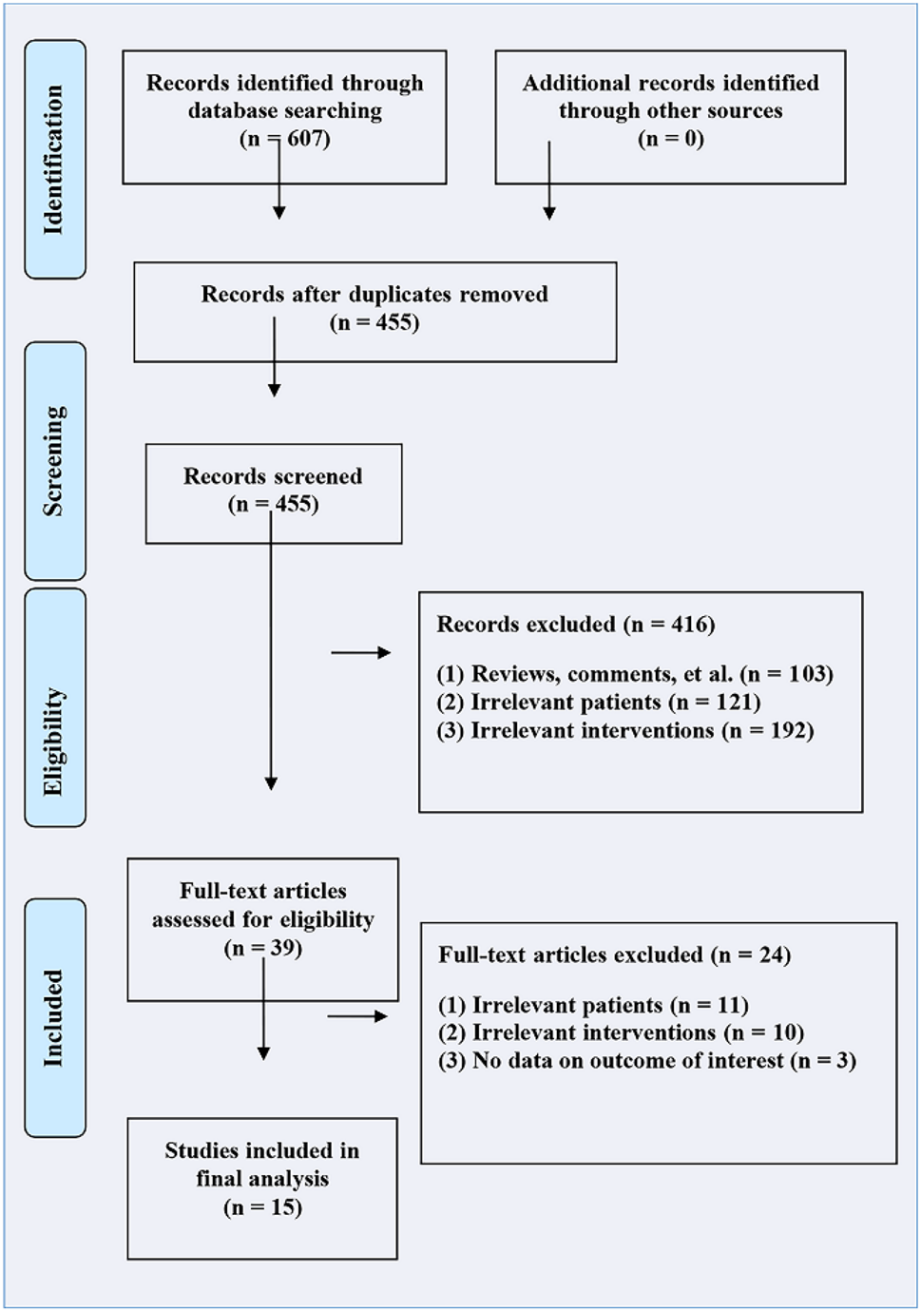
Flow diagram of study identification and selection

The inclusion criteria were strictly implemented, and 607 relevant studies were retrieved from the major databases. After excluding noncase-control studies, repeated publications, and other irrelevant studies, 39 relevant studies were initially screened. Finally, 15 papers were included after careful reading of the full text. The literature screening process and results are shown in Fig. 1, and the basic characteristics of the included studies are shown in Table 1.

**Table 1:** General characteristics of included studies

References	Type of study	Country	Publication year	OLIF/TLIF	Cases	Age (mean)	Gender (M/F)	Outcome	NOS scale
Abbasi and Murphy [10]	Retrospective	USA	2015	OLIF	28	56.1 ± 15.2	–	(1)(2)(3)	7
				TLIF	9	64.1 ± 20.9	–		
Abbasi and Grant [11]	Retrospective	USA	2018	OLIF	68	54.66 ± 16.34	35/33	(1)(2)(3)	7
				TLIF	225	59.64 ± 13.00	104/121		
Champagne et al. [12]	Retrospective	Canada	2019	OLIF	38	62	15/23	(11)	7
				TLIF	45	63	18/27		
Chen et al. [13]	Retrospective	China	2018	OLIF	34	66 ± 11	12/22	(1)(2)(5)(12)	7
				TLIF	39	66 ± 12	19/20		
Chen et al. [14]	Retrospective	China	2021	OLIF	38	61.84 ± 6.20	21/17	(1)(2)(3)(5)	7
				TLIF	40	61.15 ± 5.52	23/17		
Du et al. [15]	Retrospective	China	2021	OLIF	28	53.6 ± 6.4	16/12	(11)	8
				TLIF	37	52.8 ± 7.1	23/14		
Lee et al. [16]	Retrospective	Korea	2021	OLIF	20	68.4 ± 5.6	9/11	(1)(2)(3)(4)(5)	8
				TLIF	20	66.5 ± 6.8	8/12		
Li et al. [17]	Retrospective	China	2021	OLIF	28	57.5 ± 10.4	7/21	(1)(2)(3)(4)(5)(6)(7)(8)(11)(2)	6
				TLIF	35	59.3 ± 9.86	8/27		
Li et al. [18]	Retrospective	China	2022	OLIF	36	58.52 ± 7.26	6/30	(4)(5)(6)(8)(9)(12)	8
				TLIF	36	59.88 ± 7.04	10/26		
Mun et al. [19]	Prospective	Korea	2019	OLIF	74	64.1 ± 9.3	20/54	(1)(2)(4)(5)(6)(7)(9)(10)(11)	8
				TLIF	74	66.4 ± 10.6	24/50		
Takaoka et al. [20]	Retrospective	Japan	2021	OLIF	66	66 ± 12	28/38	(4)(11)(12)	9
				TLIF	79	71 ± 9	37/42		
Tung et al. [21]	Prospective	China	2021	OLIF	20	66.5 ± 4.2	3/17	(6)(7)(10)(11)	7
				TLIF	41	64.7 ± 5.5	6/35		
Yang et al. [22]	Random	China	2022	OLIF	60	55.5 ± 6.1	18/42	(1)(2)(3)(5)(6)	8
				TLIF	60		16/44		
Yoon et al. [23]	Retrospective	Korea	2022	OLIF	60	66.0 ± 8.4	23/37	(1)(2)(3)(6)(8)(10)(11)(12)	8
				TLIF	58	66.3 ± 9.6	19/39		
Zhao et al. [24]	Retrospective	China	2021	OLIF	46	61.7 ± 9.1	20/26	(1)(2)(3)(4)(5)(6)(8)(9)(10)(11)(12)	9
				TLIF	52	63.8 ± 10.8	21/31		

Outcomes: (1) surgery time, (2) Blood loss, (3) Hospital stay, (4) VAS, (5) ODI, (6) DH, (7) FH, (8) FSL, (9) Cage Height, (10) Fusion rate, (11) Complication, (12) Imaging indicators

*TLIF* transforaminal lumbar interbody fusion, *OLIF* oblique lumber interbody fusion, *M* male, *F* female

### Quality assessment of included literature

This study included 15 articles, of which 1 was a randomized controlled study, 2 prospective studies, and 12 retrospective studies. The NOS was used for quality assessment, of which six papers scored 8 points, two scored 9 points, eight high-quality papers, six scored 7 points, and one scored 6 points, with a total of seven medium-quality papers.

## Outcomes

### Intraoperative indicators and hospital stay

**Fig. 2:**
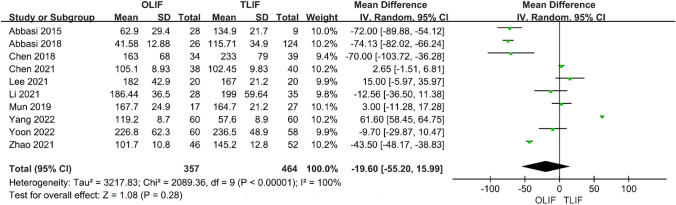
Forest plot for the surgery time

**Fig. 3:**
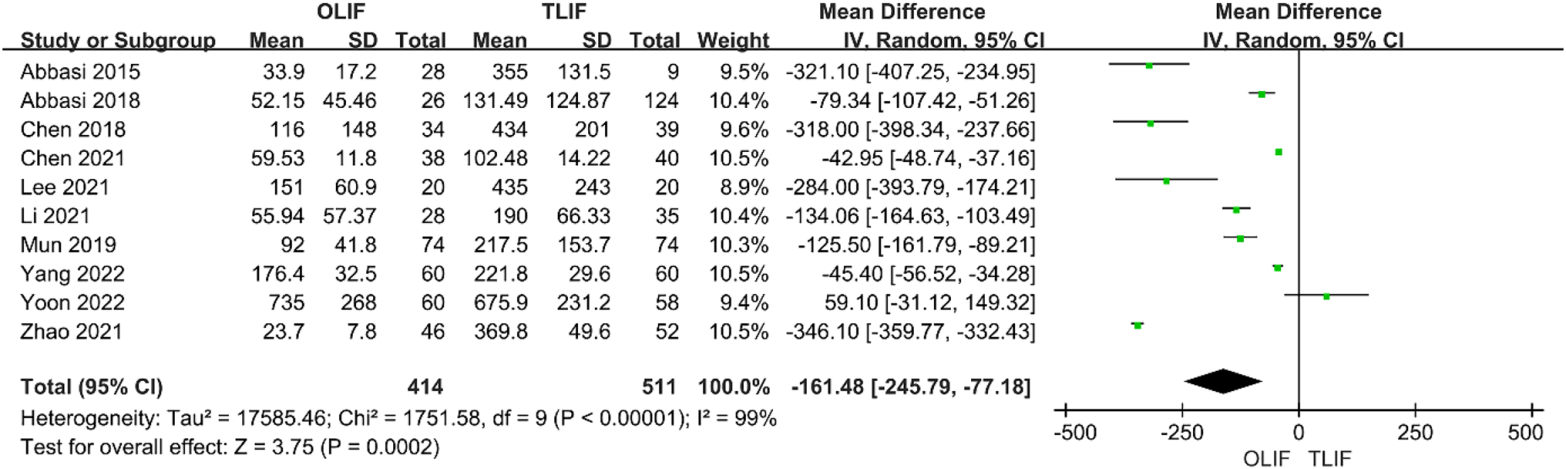
Forest plot for the blood loss

**Fig. 4:**
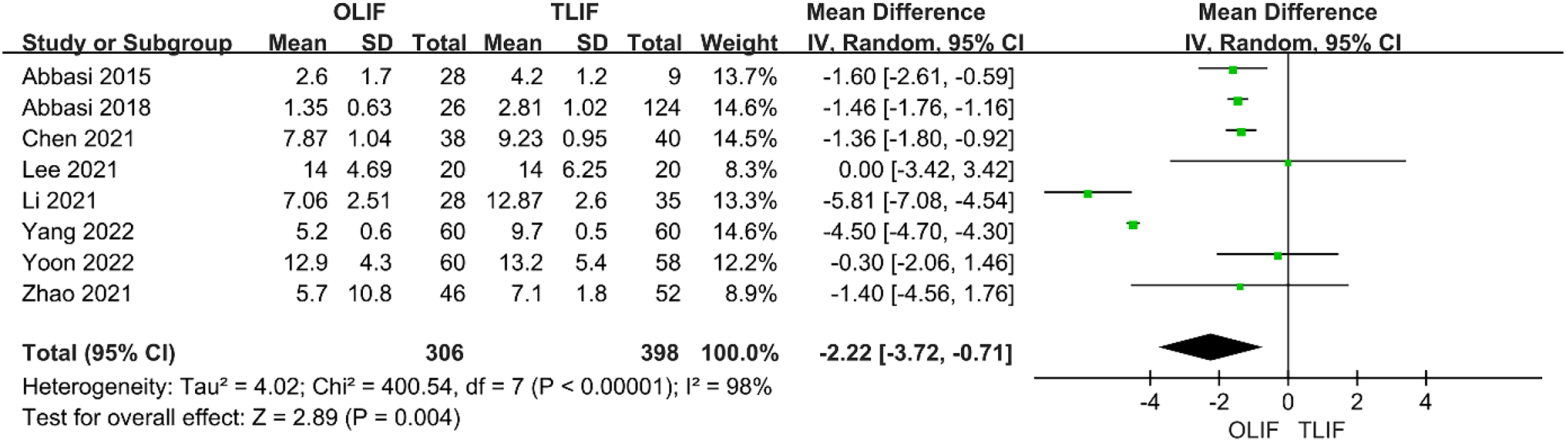
Forest plot for the hospital stay

Intraoperative indicators included surgical time and intraoperative blood loss. Ten studies compared the surgery time between OLIF and TLIF. The heterogeneity test (*I*^2^ = 100%) indicated significant heterogeneity among the studies; therefore, a random-effects model was used. The results showed that in the treatment of lumbar degenerative diseases, the operation times of the two groups were equivalent, and the difference was not statistically significant [95% CI (− 55.20 to 15.99), *P* = 0.28] (Fig. 2). There were also 10 studies comparing the intraoperative blood loss between the two groups, and the heterogeneity test *I*^2^ = 99%, indicating significant heterogeneity among the studies; thus, a random-effects model was used. The results showed that the intraoperative blood loss in OLIF was significantly lower than that in TLIF, and the difference was statistically significant [95% CI (− 245.79 to − 77.18), *P* < 0.001] (Fig. 3). Eight studies compared hospital stays between OLIF and TLIF. The heterogeneity test showed large heterogeneity among the studies (*I*^2^ = 98%, *P* < 0.001); thus, a random-effects model was used. The results showed that the hospital stay of OLIF was significantly lower than that of TLIF, and the difference was statistically significant [95% CI (− 3.72 to − 0.71), *P* = 0.004] (Fig. 4).

### Postoperative VAS and ODI

**Fig. 5:**
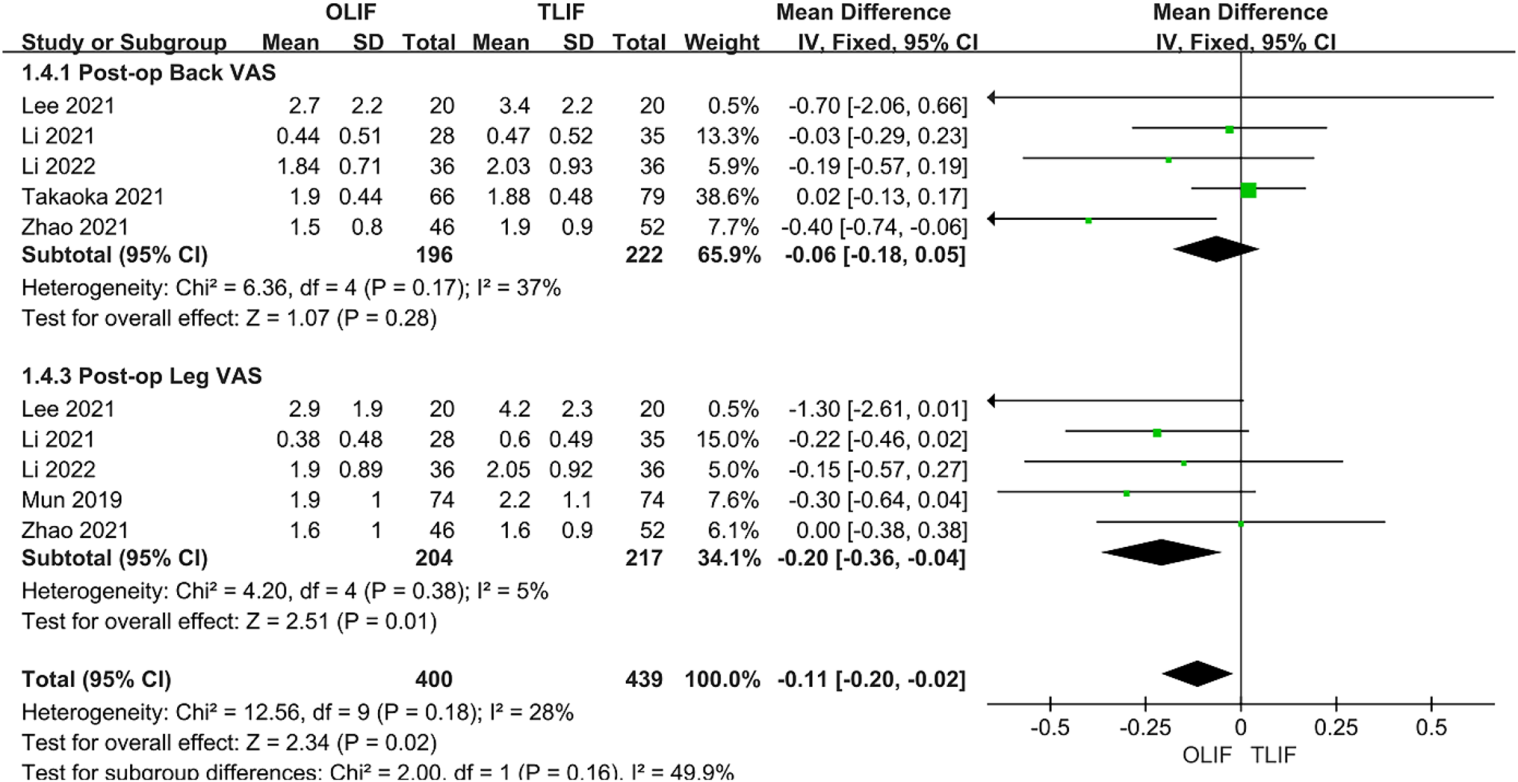
Forest plot for the VAS

**Fig. 6:**
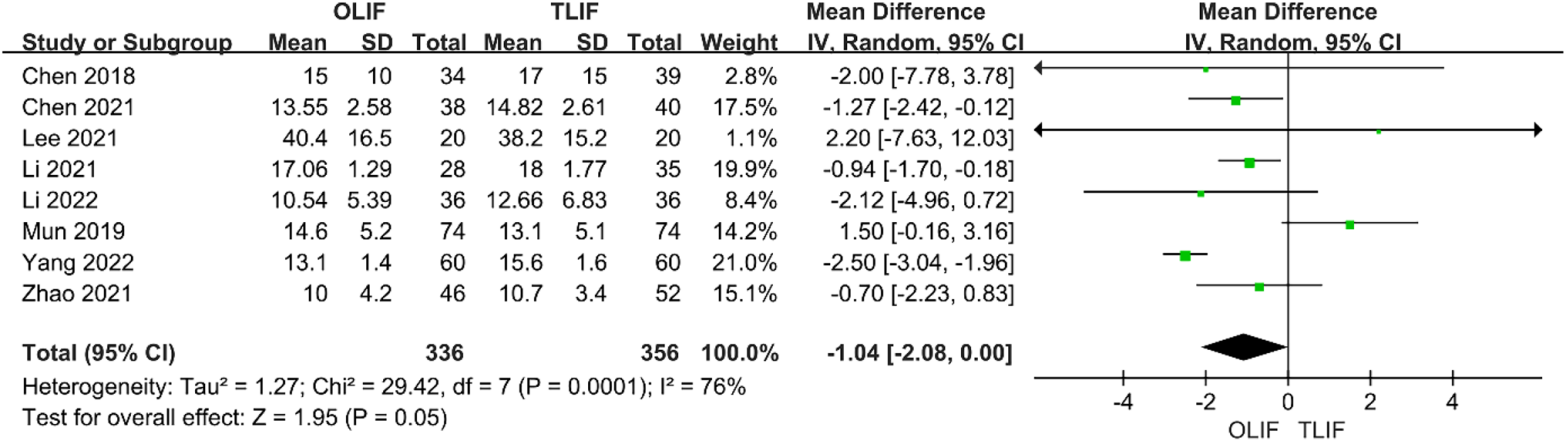
Forest plot for the ODI

A total of six papers compared VAS after OLIF and TLIF, of which five compared low back pain and six compared leg pain. Through heterogeneity analysis, we found that the heterogeneity between studies in VAS-LP was *I*^2^ = 96%, but after removing Takaoka 2021, the heterogeneity was reduced to 5%, and the overall heterogeneity between groups was *I*^2^ = 28%; thus, a fixed-effects model was used. The results showed that the efficacy of VAS-BP was similar between the two groups [95% CI (− 0.18 to 0.05), *P* = 0.28]. However, OLIF was significantly better than TLIF for VAS-LP [95% CI (− 0.36 to − 0.04), *P* = 0.0.01] (Fig. 5). A total of eight articles compared the postoperative ODI of the two surgical methods, and the heterogeneity analysis indicated *I*^2^ = 76%; thus, a random-effects model was used. The results showed that ODI after OLIF was better than after TLIF, and the difference was statistically significant [95% CI (− 2.08 to 0.00), *P* = 0.05] (Fig. 6).

### Postoperative DH, FH and FSL

**Fig. 7:**
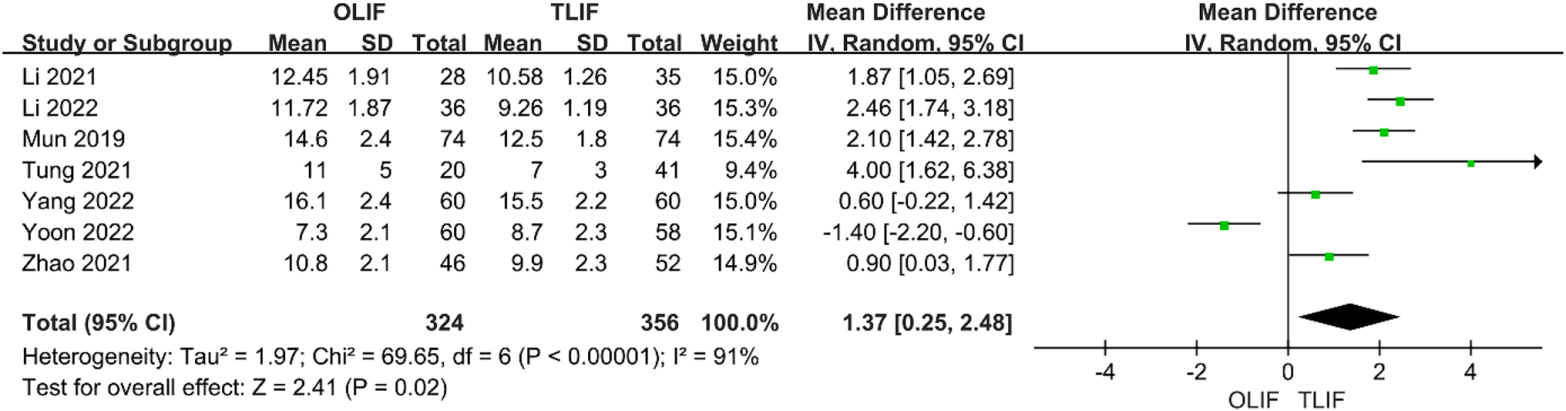
Forest plot for the DH

**Fig. 8:**
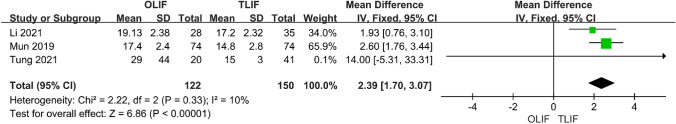
Forest plot for the FH

**Fig. 9:**
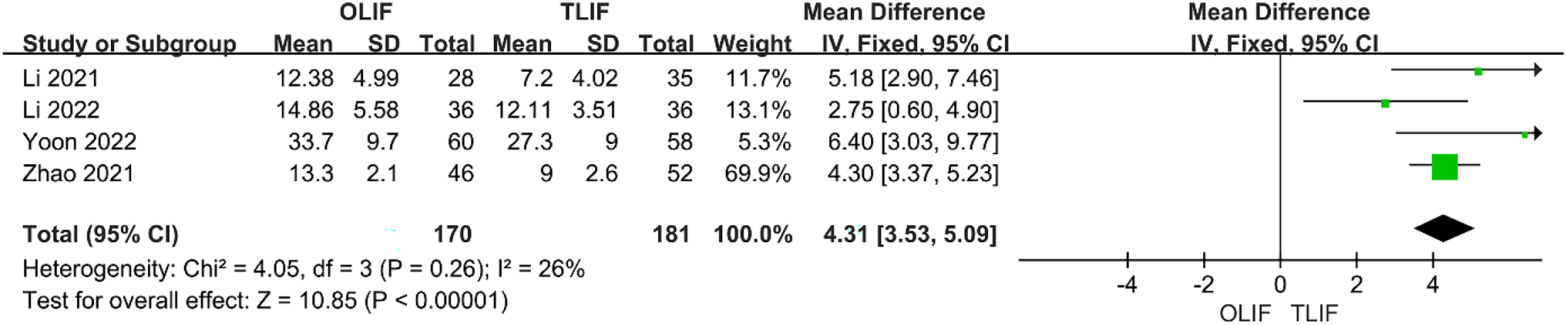
Forest plot for the FSL

Seven articles compared postoperative DH between OLIF and TLIF, and the heterogeneity test between studies (*I*^2^ = 91%) indicated that the heterogeneity was large; therefore, a random-effects model was used. The results showed that DH after OLIF was significantly higher than that after TLIF [95% CI (0.25–2.48), *P* = 0.02] (Fig. 7). Four studies compared postoperative FH, and the heterogeneity analysis indicated *I*^2^ = 65%; however, after removing Yoon 2022, it was found that the heterogeneity was reduced to 10%; therefore, a fixed-effect model was used. The results showed that OLIF was better than TLIF for FH [95% CI (1.70–3.07), *P* < 0.001], and the difference was statistically significant (Fig. 8). A total of five articles described postoperative FSL, and the heterogeneity test between studies was *I*^2^ = 77%, but after excluding the article of Tung 2021, the heterogeneity was reduced to 26%; therefore, the fixed-effect model was used. The results showed that OLIF was superior to TLIF in terms of postoperative FSL, and the difference was statistically significant [95% CI (3.53–5.09), *P* < 0.001] (Fig. 9).

### Cage height and fusion rate

**Fig. 10:**
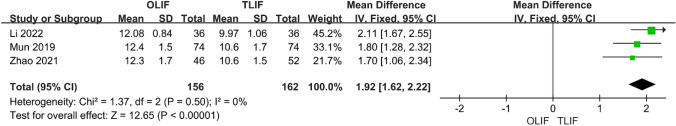
Forest plot for the Cage height

**Fig. 11:**
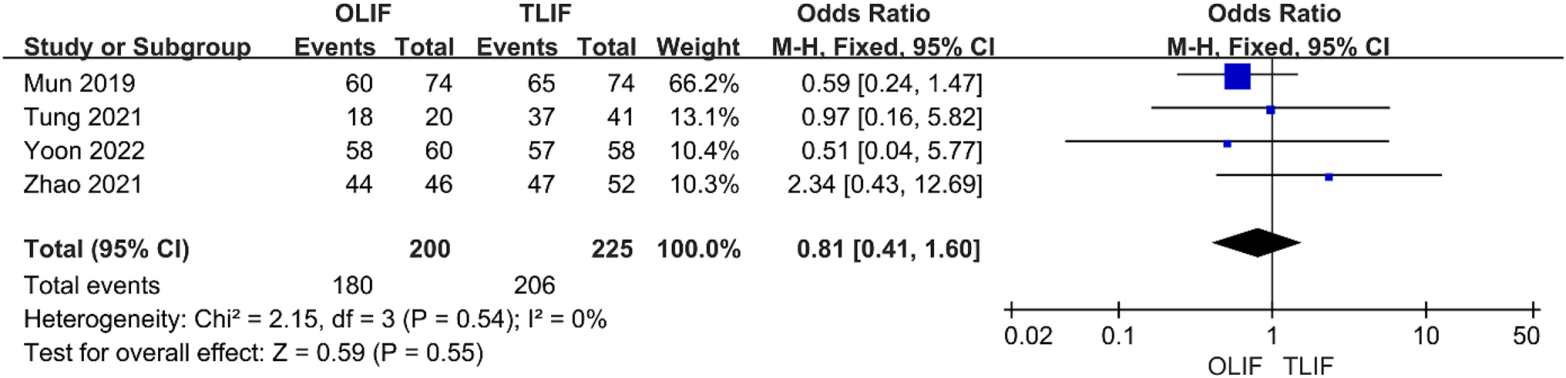
Forest plot for the Fusion rate

A total of four papers compared the height of the OLIF and TLIF cages, and the heterogeneity test showed *I*^2^ = 71%. After excluding Yoon 2022, the heterogeneity was reduced to 0%, and the fixed-effect model was used. The results show that the cage height of OLIF was significantly higher than that of TLIF, and the difference was statistically significant [95% CI (1.62–2.22), *P* < 0.001] (Fig. 10). There were four articles comparing the postoperative fusion rate between the two procedures, and the heterogeneity between the studies was low (*I*^2^ = 0%); therefore, a fixed-effect model was used. The results showed no significant difference in postoperative fusion rates between the two procedures [95% CI (0.41–1.60), *P* = 0.55] (Fig. 11).

### Complications and imaging indicators

**Fig. 12:**
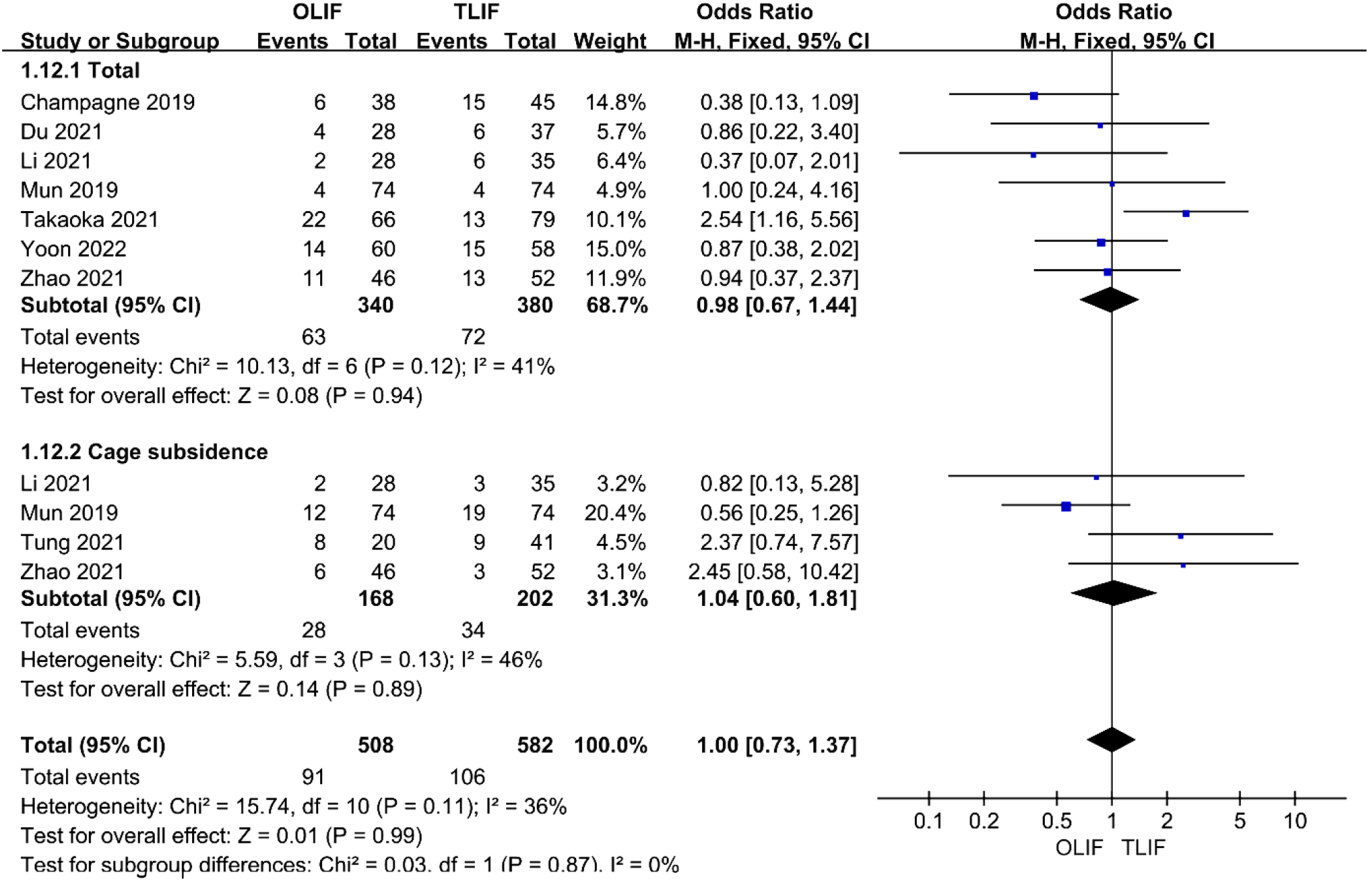
Forest plot for the complications

**Fig. 13:**
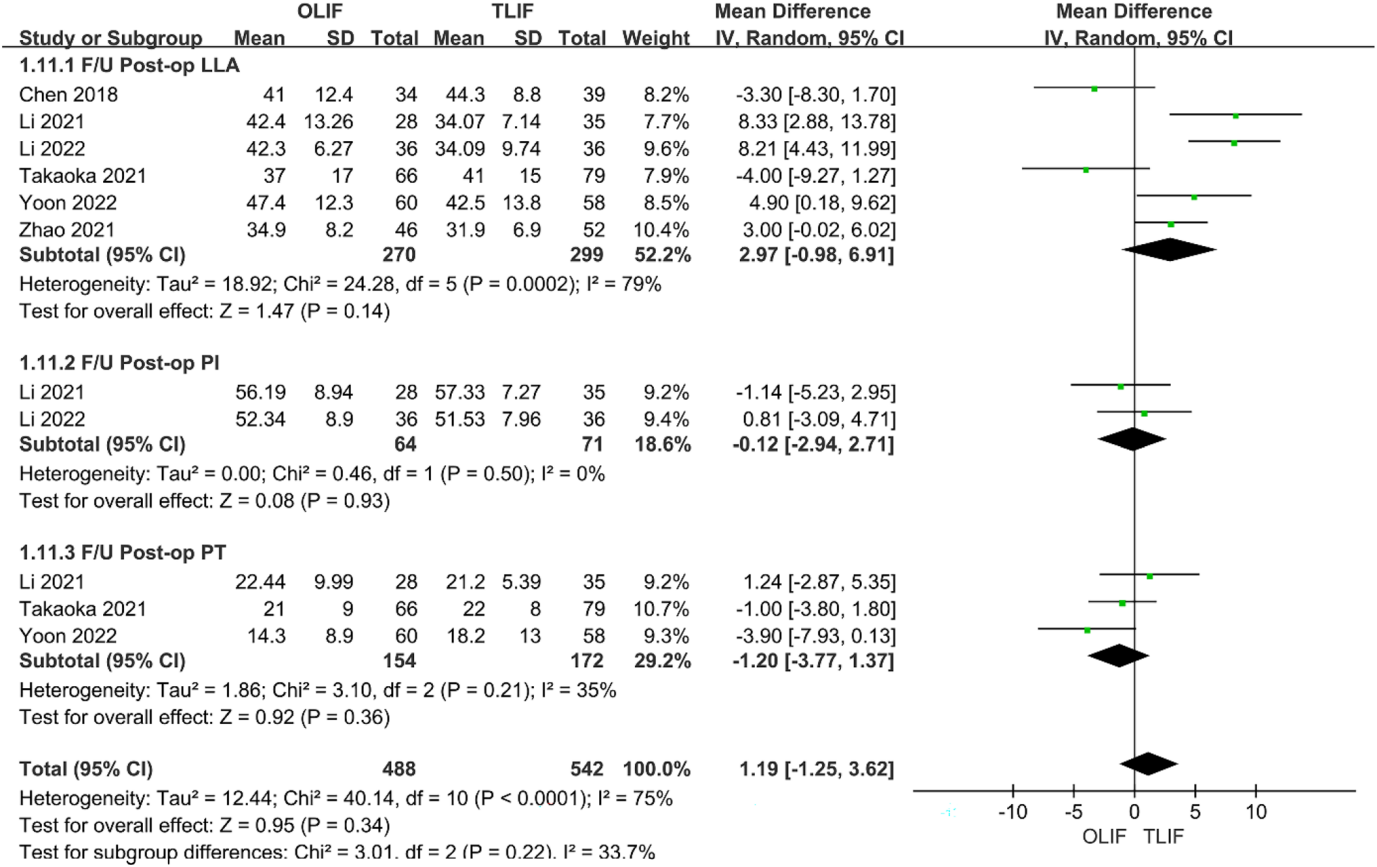
Forest plot for the imaging indicators

Seven and four articles compared postoperative complications and cage subsidence, respectively. The heterogeneity test between each group was *I*^2^ = 36%; therefore, a fixed-effect model was used. The results showed that there was no statistical difference between the two procedures [95% CI (0.67–1.44), *P* = 0.94; 95% CI (0.60–1.81), *P* = 0.89] (Fig. 12). In the present study, we found that the most common complication of OLIF was postoperative transient new-onset paresthesia, followed by endplate injury, whereas the most common complication of TLIF was dural sac injury, followed by nerve injury and postoperative wound infection. In terms of postoperative imaging indicators, such as lumbar lordosis angle (LLA), pelvic incidence (PI) and pelvic tilt (PT), there was overall heterogeneity between the groups (*I*^2^ = 75%); therefore, a random effect model was used. The results showed no statistically significant difference in all indicators [95% CI (− 0.98 to 6.91), *P* = 0.14], [95% CI (− 2.94 to 2.71), *P* = 0.93], and [95% CI (− 3.77 to 1.37), *P* = 0.36] (Fig. 13).

### Publication bias and sensitivity analysis

**Fig. 14:**
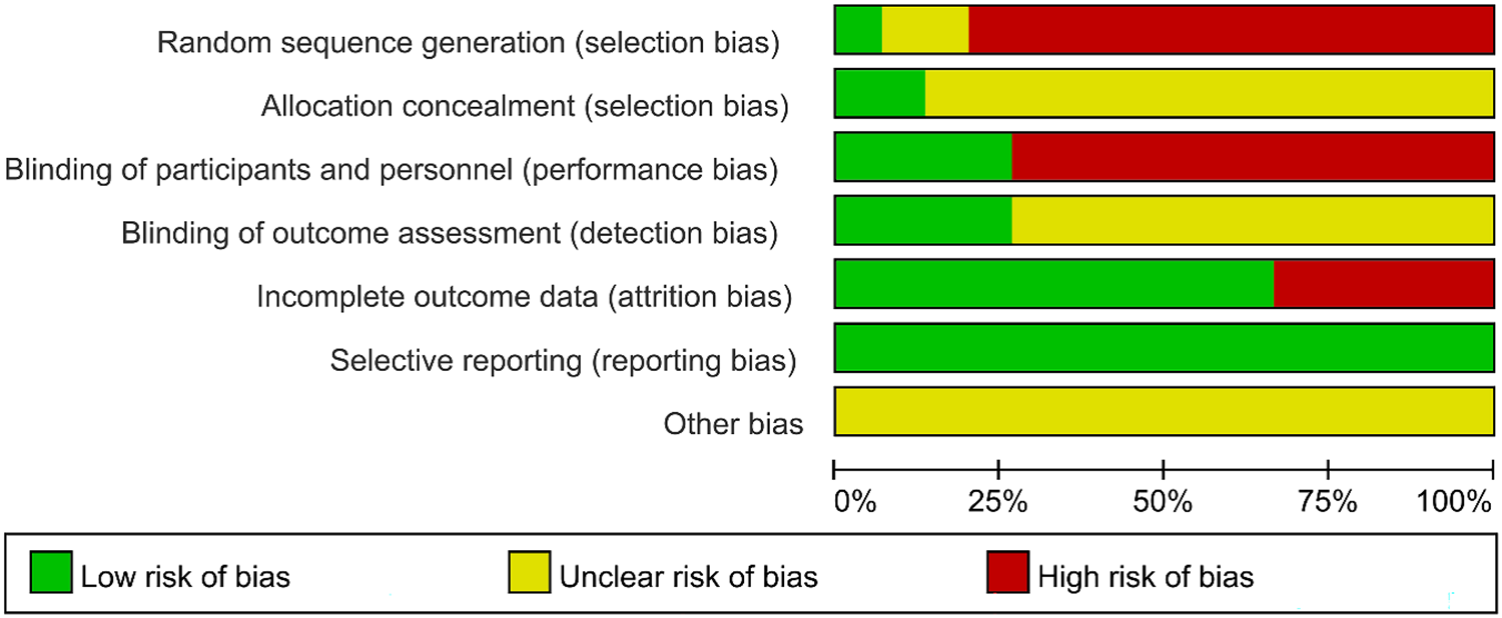
Overall risk of bias assessment of the studies included in the present meta‑analysis

**Fig. 15:**
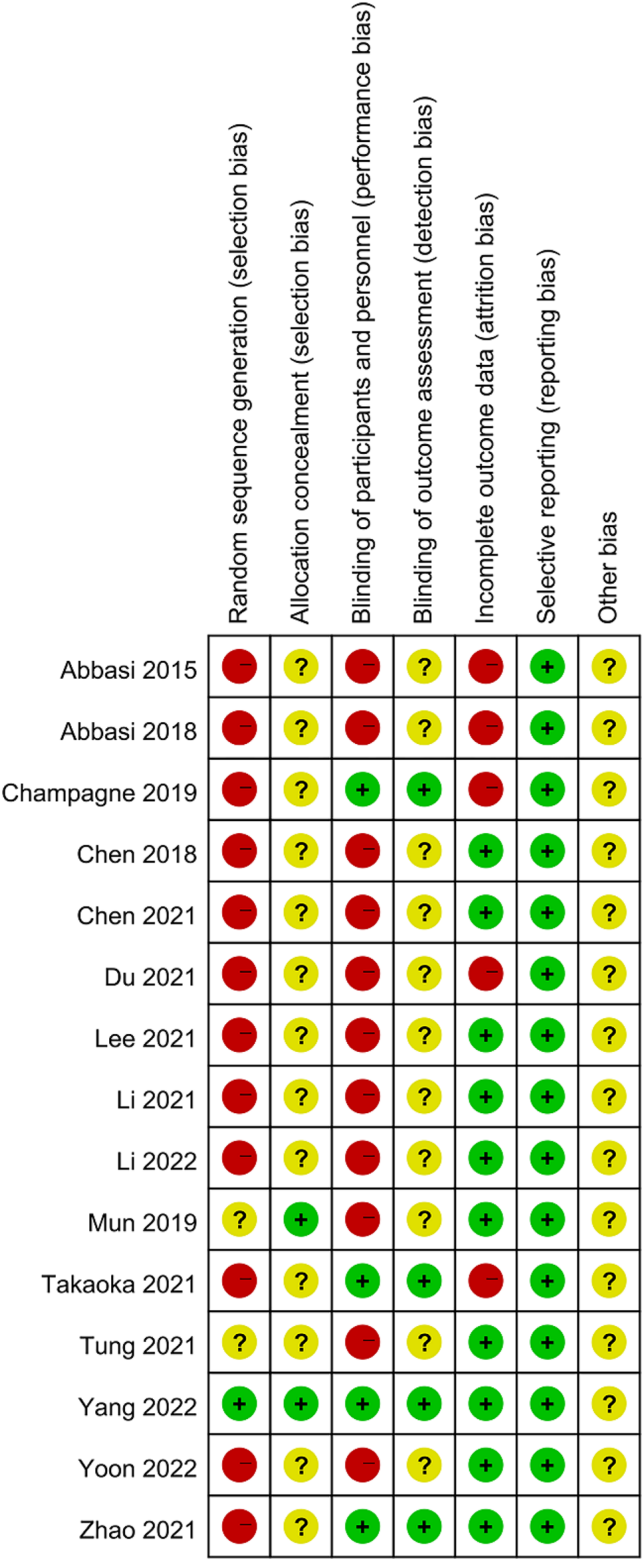
Risk of bias assessment of the specific studies included in the present meta‑analysis

**Fig. 16:**
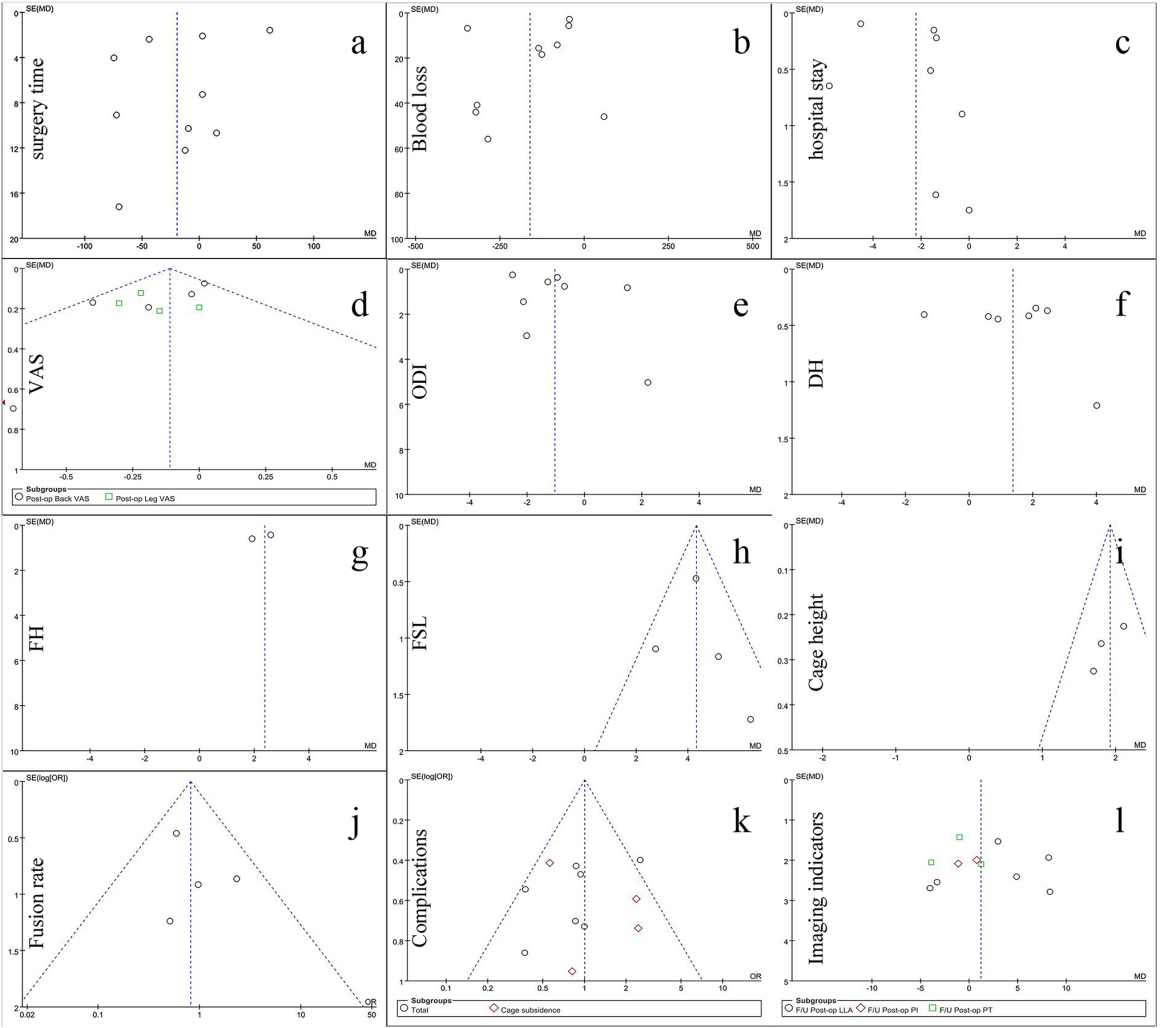
Funnel plots created to assess publication bias for **a** surgery time, **b** Blood loss, shoulder and hand score, **c** Hospital stay, **d** VAS **e** ODI, **f** DH, **g** FH, **h** FSL, **i** Cage Height, **j** Fusion rate, **k** Complications, **l** Imaging indicators

A total of 12 outcome indicators, including surgery time, intraoperative blood loss, hospital stay, VAS, ODI, DH, FH, FSL, cage height, fusion rate, complications, and imaging indicators, were analyzed using the Review manager 5.4 statistical software for publication bias analysis (Figs. 14, 15). The results showed that the funnel plots were symmetrical, suggesting that there was no obvious publication bias (Fig. 16). Among the outcome indicators, there was a large heterogeneity (*I*^2^ > 50%) among the studies on surgery time, intraoperative blood loss, hospital stay, VAS, ODI, DH, FH, FSL, cage height, and imaging indicators. The heterogeneity of some outcome indicators was reduced (*I*^2^ < 50%) after excluding some papers, and the heterogeneity of the remaining indicators showed no directional changes after excluding the included papers individually, indicating that the results were relatively stable. Furthermore, the included studies did not classify data according to age and sex; therefore, subgroup analyses could not be performed.

## Discussion

Low back pain caused by lumbar degenerative diseases mainly refers to pain between the ribs and buttocks caused by lumbar structures (vertebral bodies, intervertebral discs, and muscles) [25]. The lifetime prevalence of an individual is as high as 80%, and an increasing number of individuals require surgical treatment [26]. The main purpose of spine surgery is to relieve pain and improve function and quality of life [27]. Restoring the height of the intervertebral space and the stability of the spine through intervertebral fusion has always been considered an effective treatment for lumbar degenerative diseases. Currently, the widely used surgical methods include PLIF and TLIF, both of which can achieve good clinical efficacy [28]. However, many studies have shown that TLIF can avoid nerve and dural sac injury and has the advantages of shorter operation time and less intraoperative blood loss [29]. OLIF is a popular minimally invasive retroperitoneal lumbar interbody fusion surgery that also has the advantages of less intraoperative trauma and faster recovery [30]. Therefore, this meta-analysis aimed to compare the efficacy of OLIF and TLIF in the treatment of lumbar degenerative diseases.

In this study, we found that OLIF has obvious advantages in terms of intraoperative blood loss and hospital stay, which is related to the entry of OLIF from the natural gap between the psoas muscle and blood vessels. Related operations, such as paravertebral muscle dissection and intervertebral foramen opening in the posterior approach of TLIF, are avoided, thereby reducing the amount of intraoperative blood loss and hospitalization time, which is consistent with the results of Yang et al. [22]. Some scholars believe that the large amount of intraoperative blood loss in TLIF may lead to an unclear intraoperative visual field and prolong the surgery time. However, in this study, no statistically significant difference was found between the two in terms of surgery time.

In terms of VAS, this study found that the degree of VAS-BP was approximately the same at the last follow-up. Many studies have shown that both OLIF and TLIF can relieve low back and leg pains [31]. However, in this study, we found that OLIF was superior to TLIF for VAS-LP. This may be because the indirect decompression properties of OLIF avoid direct nerve root traction, thereby reducing pain symptoms in the leg [32]. Gagliardi et al. believed that both direct decompression and indirect decompression can effectively reduce pain and dysfunction caused by lumbar spine disease [33] and found that both OLIF and TLIF can increase the DH and FH of the surgical segment on the imaging data, proving that both can achieve good postoperative outcomes. However, OLIF has obvious advantages over TLIF in postoperative DH and FH because the surgical method of OLIF involves the implantation of a larger cage in the intervertebral disc of the surgical segment to achieve indirect decompression [17]. In this study, we found that the OLIF-implanted cage size was significantly larger than that of TLIF. This may be the natural advantage of the OLIF oblique lateral approach, and the operator has a better field of view and space to implant a large cage from the front to achieve a better FSL [13]. This finding is consistent with the results of the present study. In theory, better FSL, DH, and FH should have better postoperative functional efficacy. In this study, we found that OLIF was slightly better than TLIF in terms of postoperative ODI, but the advantage was not obvious. This may be related to the fact that although OLIF indirectly decompresses the nerve root by increasing DH and FH, it does not open the spinal canal and cannot change the cross-sectional area of the spinal canal; thus, patients with severe spinal stenosis may not be suitable for OLIF [34].

Although OLIF uses a larger cage, there was no statistically significant difference in the interbody fusion rates between OLIF and TLIF. The intervertebral fusion rate is affected by many factors, such as intervertebral space preparation, endplate grading, and cage material [35]. However, in general, both can achieve a better fusion rate. This study found no significant difference in postoperative complications between the two groups. Although OLIF avoids posterior nerve root injury, cerebrospinal fluid leakage, and other related complications compared with TLIF [36], there are also related complications, such as postoperative intestinal obstruction, vascular injury, sympathetic nerve, and visceral injury [37]. However, the overall complication rate is low and serious complications are rare [38]. Ureteral injury is one of the serious complications of OLIF, which is related to the need to push the peritoneum forward to expand the field of view during the surgical approach, and its early diagnosis is difficult, and the treatment is more troublesome [39]. The fusion cage subsidence rates in OLIF and TLIF were 16.7% and 16.8%, respectively, and there was no statistically significant difference. Wu et al. believed that after the patient walked down the ground after surgery, the cage could be slightly settled by gravity to fit the endplate better, thereby accelerating fusion [40]. However, cage subsidence is fatal to OLIF because OLIF itself achieves indirect decompression of the nerve root by increasing the DH and FH through a large cage, and cage subsidence will inevitably cause the shortening of DH and FH, resulting in an unsatisfactory decompression effect.

The balance of the spine and sagittal plane of the pelvis is an important factor in the transmission of force lines in the spine. If the angle of the sagittal plane of the spine cannot maintain the balance of the spine, the human body will compensate by tilting the pelvis forward and backward [41]. In this study, we found that although both OLIF and TLIF can improve LLA, there was no significant difference in LLA, PI, and PT after surgery, indicating that both can better maintain spinal stability, thereby avoiding the pelvic compensation mechanism.

In this study, we found that both OLIF and TLIF can effectively improve the symptoms of low back pain in lumbar degenerative diseases. However, OLIF is superior to TLIF in leg pain relief and has the advantages of less intraoperative blood loss and shorter hospital stays. In the postoperative functional score, indirect decompression of OLIF can achieve a very good curative effect and even has certain advantages over TLIF. The rate of intervertebral fusion was the same, and there was no significant difference in postoperative complications and sagittal indexes of the spine and pelvis. Therefore, we believe that OLIF is superior to TLIF when fusion surgery is required to treat degenerative lumbar spine diseases.

## Limitation

This meta-analysis had the following limitations: (1) there were insufficient randomized controlled trials included in this analysis, and the level of evidence was not high; (2) the specific operation in surgery might be slightly different in different studies, and the surgical results were affected by many variables, such as anatomy, and the final conclusion might be slightly different from the real results; (3) among all outcome indicators, at most 10 and at least 3 articles described the same indicator, the heterogeneity between studies increased slightly, and the difference in the final follow-up time between the various papers might have a certain impact on the results, and we have no way of knowing the longer-term efficacy; and (4) due to the lack of mention of the economic cost, difference between pre and postoperative scores, cage materials, and renovation rate of the two surgeries in the included literature, this study could not compare those factors. There are few studies included in this study, and the final results may still be different from the actual situation. More studies with larger sample sizes are needed for verification. We look forward to more clinical case–control studies on the treatment of lumbar degenerative diseases in the future to reduce bias and draw more realistic and reliable conclusions.

## Data Availability

The datasets used and/or analyzed during the current study are available from the corresponding author upon reasonable request.
